# Simple Accurate Verification of Enthalpy‐Entropy Compensation and Isoequilibrium Relationship

**DOI:** 10.1002/cphc.202100431

**Published:** 2021-07-20

**Authors:** Ronald Griessen, Bernard Dam

**Affiliations:** ^1^ Condensed Matter Physics Faculty of Sciences VU University Amsterdam De Boelelaan 1081 1081 HV Amsterdam, The Netherlands; ^2^ Materials for Energy Conversion and Storage Department of Chemical Engineering Faculty of Applied Sciences Delft University of Technology Van der Maasweg 9 2629 HZ Delft, The Netherlands

**Keywords:** thermodynamics, artifacts, statistics, simulations, isokinetic temperature

## Abstract

In many experimental investigations of thermodynamic equilibrium or kinetic properties of series of similar reactions it is found that the enthalpies and entropies derived from Van ′t Hoff or Arrhenius plots exhibit a strong linear correlation. The origin of this Enthalpy‐Entropy compensation, which is strongly related to the coalescence tendency of Van ′t Hoff or Arrhenius plots, is not necessarily due to a physical/chemical/biological process. It can also be a merely statistical artefact. A new method, called Combined *K‐CQF* makes it possible **both** to quantify the degree of coalescence of experimental Van ‘t Hoff lines **and** to verify whether or not the Enthalpy‐Entropy Compensation is of a statistical origin at a desired confidence level. The method is universal and can handle data sets with any degree of coalescence of Van ‘t Hoff (or Arrhenius) plots. The new method requires only a standard least square fit of the enthalpy*ΔH* versus entropy *ΔS* plot to determine the two essential dimensionless parameters *K* and *CQF*. The parameter *K* indicates the position (in inverse temperature) of the coalescence region of Van ‘t Hoff plots and *CQF* is a quantitative measure of the smallest spread of the Van ‘t Hoff plots. The position of the (*K, CQF*) couple with respect to universal confidence contours determined from a large number of simulations of random Van ‘t Hoff plots indicates straightforwardly whether or not the *ΔH‐ΔS* compensation is a statistical artefact.

## Graphical abstract

**Entropy‐Enthalpy compensation**: Two dimensionless parameters *K* and *CQF* characterize quantitatively the coalescence of the Van‘t Hoff plots for similar reactions. *K* is related to the temperature at which the coalescence occurs and *CQF* is the Compensation Quality Factor, which is unity for perfect coalescence. By comparing measured (*K*, *CQF*) to universal confidence contours determined from a very large number of random simulations for *N* samples it is verified whether an Enthalpy‐Entropy Compensation is a real effect at a chosen confidence level. The same scheme is used for Arrhenius plots in kinetic studies.

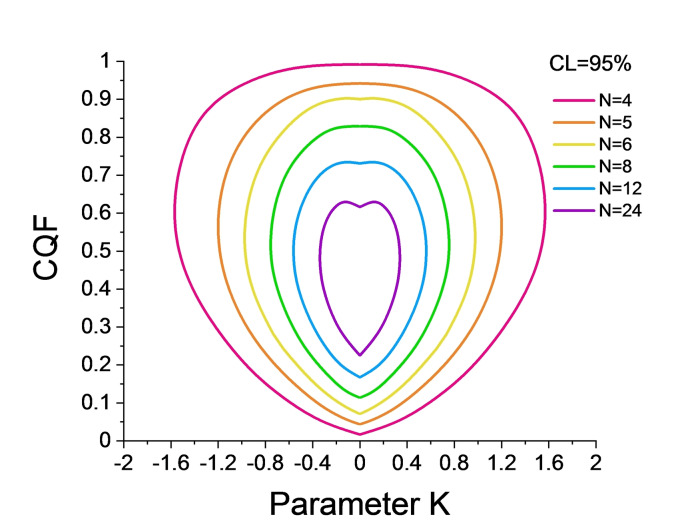

## 1 Introduction

There is a longstanding debate in the literature regarding the physical basis of the so‐called enthalpy‐entropy compensation (*EEC*), which is observed in a wide range of fields in chemistry[^1^, ^2^, ^3^, ^4^, ^5^, ^6^, ^7^, ^8^], biology[^9^, ^10^, ^11^, ^12^, ^13^, ^14^, ^15^] and solid‐state physics[^16^, ^17^, ^18^, ^19^] . *EEC* describes a linear relation between two thermodynamic parameters ‐ the enthalpy Δ*H* and entropy Δ*S* ‐ of a series of similar reactions. Examples include the denaturation of closely related proteins, reactions in slightly varying solvents, catalysis or reactions of hydrogen with metal alloys.^[20]^ While there is a wealth of data it is unclear which Δ*H*‐Δ*S* correlations are strong enough to pursue a quest for the scientific basis of the related enthalpy‐entropy compensation. In many cases Δ*H_i_* and Δ*S_i_* of a series of *N* samples (1≤*i*≤*N*), are determined from the slope and intercept of their Van ‘t Hoff plots. *EEC* is traditionally characterized by the so‐called compensation temperature *T_comp_* =d*ΔH*/d*ΔS*, which is the slope of a linear fit to the Δ*H_i_* versus Δ*S_i_* plot. Interestingly, many compensation effects have a *T_comp_* close to the harmonic mean of the experimental temperature, *T_hm_* 1

(1) $$T_{hm}={\left(\frac{1}{M}\sum_{j=1}^{M}\frac{1}{T_{j}}\right)}^{-1}$$

where *T_j_* with *j*=1,…*M*, is the temperature of the *j‐th* measurement at which the equilibrium pressures *P_i_* (*T_j_*) of the *N* samples are determined. Hence, several authors have claimed that when *T_comp_* ≈*T_hm_*, statistical and/or experimental errors are causing these compensation effects.[^21^, ^22^, ^23^, ^24^, ^25^, ^26^, ^27^, ^28^, ^29^, ^30^] Krug et al.[^31^, ^32^] proposed an approximate statistical test to verify whether the harmonic mean experimental temperature *T_hm_* falls outside the interval [*T_comp_*‐*t*σ, *T_comp_* +*t*σ ], in order to verify the physical nature of the effect. Here, σ is the standard error in *T_comp_* and the Student's *t‐value* depends on the chosen confidence level and the number of samples, i. e. the number of (*ΔH,ΔS*) data pairs. However, this approach does not predict the degree of coalescence of Van ‘t Hoff lines observed near *T_comp_*.

In our previous article we introduced a Compensation Quality Factor (*CQF*) as a measure of the isoequilibrium relationship. If *CQF*>0.9, a compensation effect with a high degree of coalescence can be inferred. In that case, the location of *T_comp_* is hardly relevant. However, strong enthalpy‐entropy compensation effects are observed in the absence of a strong iso‐equilibrium effect, i. e. at *CQF*<0.9. In that case, *T_comp_* in relation to *T_hm_* is of relevance to decide whether we are dealing with a compensation effect of a true physical nature.

The purpose of the present article is an essential improvement of the method described in our previous article^[33]^ as it takes explicitly the relation between *T_comp_* and *T_hm_* into account. It aims to provide a very simple, yet accurate verification scheme addressing the nature of *both* the Enthalpy‐Entropy Compensation *and* the Isoequilibrium Relationship.

## 2 The Two Parameters of the Model

**Figure 1:**
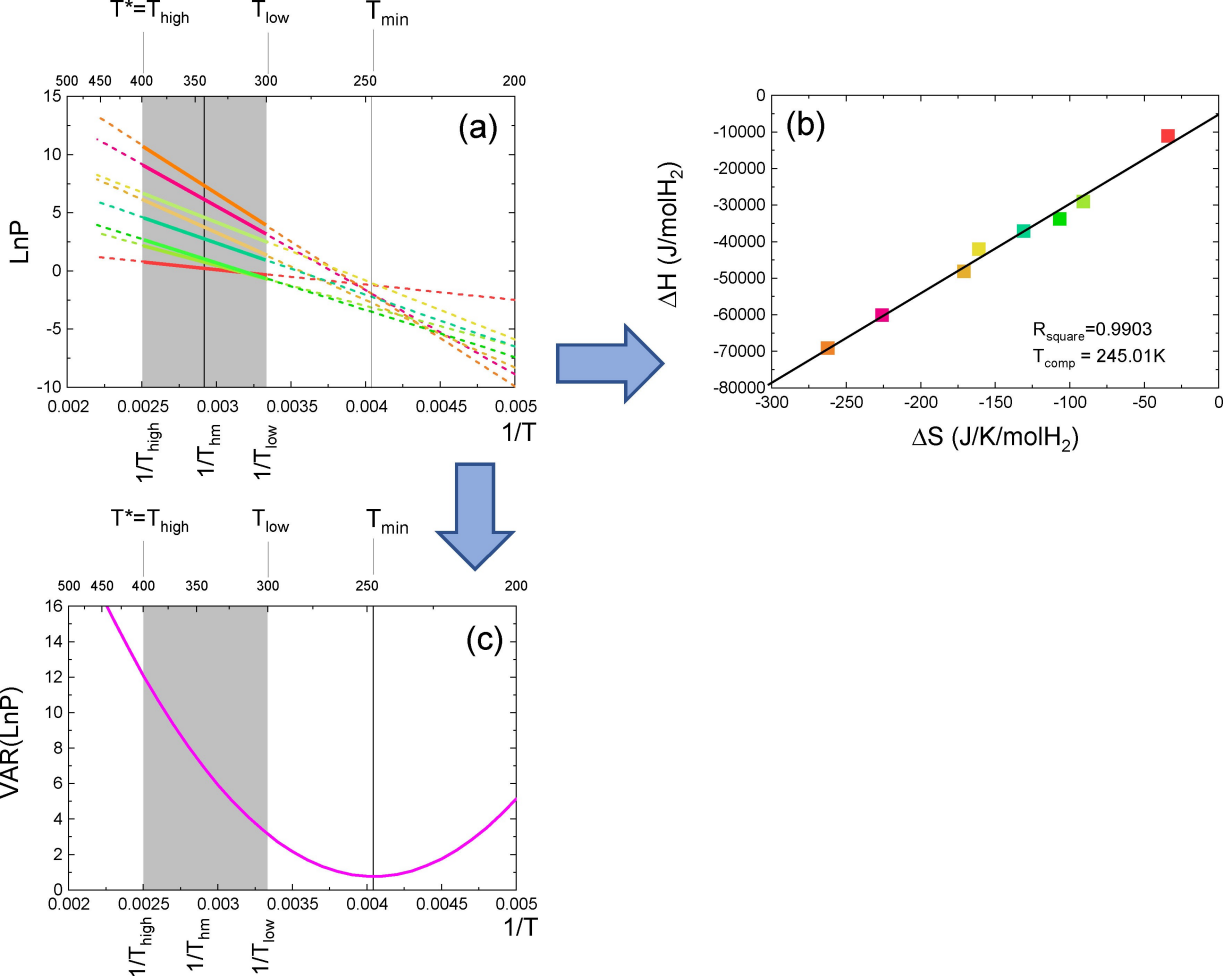
(a): Van ‘t Hoff plots for the *N*=8 samples of a hypothetical set of measurements. For this example *T_low_* =300 K, *T_high_* =400 K and from Eq.(1) *T_hm_* =342.86 K ; (b): Enthalpy versus entropy plot using the *ΔH* and *ΔS* determined from the Van ‘t Hoff plots in panel (a) by means of Eq.(2). The slope of the fitted straight line *T_comp_* =245.01 K lays outside the chosen temperature range [300 K, 400 K], which is indicated as a grey rectangle. The coefficient of determination is *R_square_* is 0.9903; (c):Inverse temperature variation of the *LnP_Spread* of the Van ‘t Hoff plots in panel (a) calculated by means of Eq. (4). The minimum spread occurs at *T_min_* =247.41 K.

By means of the hypothetical example shown in Figure 1 we introduce all the relevant input parameters of our model. In the spirit of our previous article^[33]^ we consider the absorption of hydrogen in a set of *N*=8 samples. From the hypothetic hydrogen pressure‐composition isotherms the equilibrium plateau pressures *P_i_* arising from the coexistence of a dilute and a concentrated hydride phase are obtained as a function of temperature within the temperature interval [*T_low_, T_high_*].

Using the Van ‘t Hoff equation

(2) $$ln\left(\frac{P_{i}}{P_{0}}\right)=\frac{{\Delta H}_{i}}{RT}-\frac{{\Delta S}_{i}}{R}$$

where *P_0_* is the pressure at standard conditions, *R* the gas constant, we determine the enthalpies Δ*H_i_* and entropies Δ*S_i_* for the *N* samples (1≤*i*≤*N*). Both Δ*H_i_* and Δ*S_i_* are expressed per mole H_2_. The slope of the Δ*H_i_* versus Δ*S_i_* plot in Figure 1b, which is by definition the so‐called compensation temperature, is

(3) $$T_{comp}=\frac{d\Delta H}{d\Delta S}=245.01\;K$$

The coefficient of determination of the fit *R_square_* =0.9903 is close to unity. An extrapolation of the Van ‘t Hoff plots to lower temperatures indicates that there is a certain degree of coalescence near 250 K. A precise determination of the temperature *T_min_* at which the smallest spread in *LnP* (indicated by *LnP_Spread*) occurs is obtained by plotting4

(4) $$\;LnP\_Spread\left(T\right)=\sqrt{VAR\left(LnP\left(T\right)\right)}=\;\;=\sqrt{\frac{1}{N-1}\sum_{i=1}^{N}{\left(LnP_{i}\left(T\right)-<LnP\left(T\right)>\right)}^{2}}$$

as a function of the inverse temperature. The smallest value of the *LnP_Spread* and the temperature *T_min_* at which it occurs characterize the coalescence of the Van ‘t Hoff plots. In Ref. [33] we showed that the quality of the coalescence can be quantitatively characterized with the Compensation Quality Factor *CQF* 5

(5) $$CQF=1-\sqrt{\frac{Min\left(LnP\_Spread\right)}{Max\left(LnP\_Spread\right)}}=1-\sqrt{\frac{LnP\_Spread\left(T_{min}\right)}{LnP\_Spread\left(T{}^{*}\right)}}$$

where T* is the temperature at which the **measured** *LnP_Spread* is largest. We have T*=T_low_ if 1/*T_min_* is closer to 1/*T_high_* or T*=T_high_ if 1/*T_min_* is closer to 1/*T_low_*. In the example of Figure 1, T*=400 K and *T_min_* =247.41 K. Interestingly, both *T_min_* and *CQF* can be expressed in terms of the two fit parameters of the Δ*H_i_* versus Δ*S_i_* plot, *T_comp_* and *R_square_* by means of

(6) $$T_{min}=\frac{T_{comp}}{R_{square}}$$

and

(7) $$CQF=1-\sqrt{\frac{1-R_{square}}{\left(\frac{1}{R_{square}}\right){\left(\frac{T_{comp}}{T{}^{*}}\right)}^{2}-2\left(\frac{T_{comp}}{T{}^{*}}\right)+1}}$$

*CQF* is equal to unity for a perfect coalescence. A vanishing *CQF* corresponds to a situation where all Van ‘t Hoff lines are parallel. As by definition *CQF* is dimensionless and involves only ratios of temperatures, it is a good parameter to compare data of different studies.

As a second step we need to characterize the position of the coalescence. We define an additional dimensionless parameter8

(8) $$K=\frac{\frac{1}{T_{hm}}-\frac{1}{T_{min}}}{\frac{1}{2}\left(\frac{1}{T_{low}}-\frac{1}{T_{high}}\right)}$$

which measures the location of coalescence with respect to the average temperature *T_hm_*. The denominator in Eq.(8) (which is related to the experimental range of temperatures [*T_low_, T_high_*]) is chosen in such a way that *K*=−1 when *T_min_=T_low_* and *K*=1 when *T_min_=T_high_*. The parameter K involves only inverse temperatures as van ‘t Hoff plots are linear as a function of 1/*T*. For the data in Figure 1, *K*=−2.700 and *CQF*=0.749. The relatively low value of *CQF*, reflects a relatively poor coalescence of the Van ‘t Hoff lines at *T_min_* in Figure 1a. This illustrates that the high value *R_square_* =0.9903 for the EEC in Figure 1b is not a good measure of the compensation quality. On the other hand, in our previous analysis^33^we showed that the *CQF* is high enough to assume a physical nature of the EEC at a confidence level of ∼98 %. In the next paragraph, we re‐evaluate this statement, now taking into account both K and *CQF*.

## 3 Simulations of Enthalpy‐Entropy Compensation and Isoequilibrium Relationship

**Figure 2:**
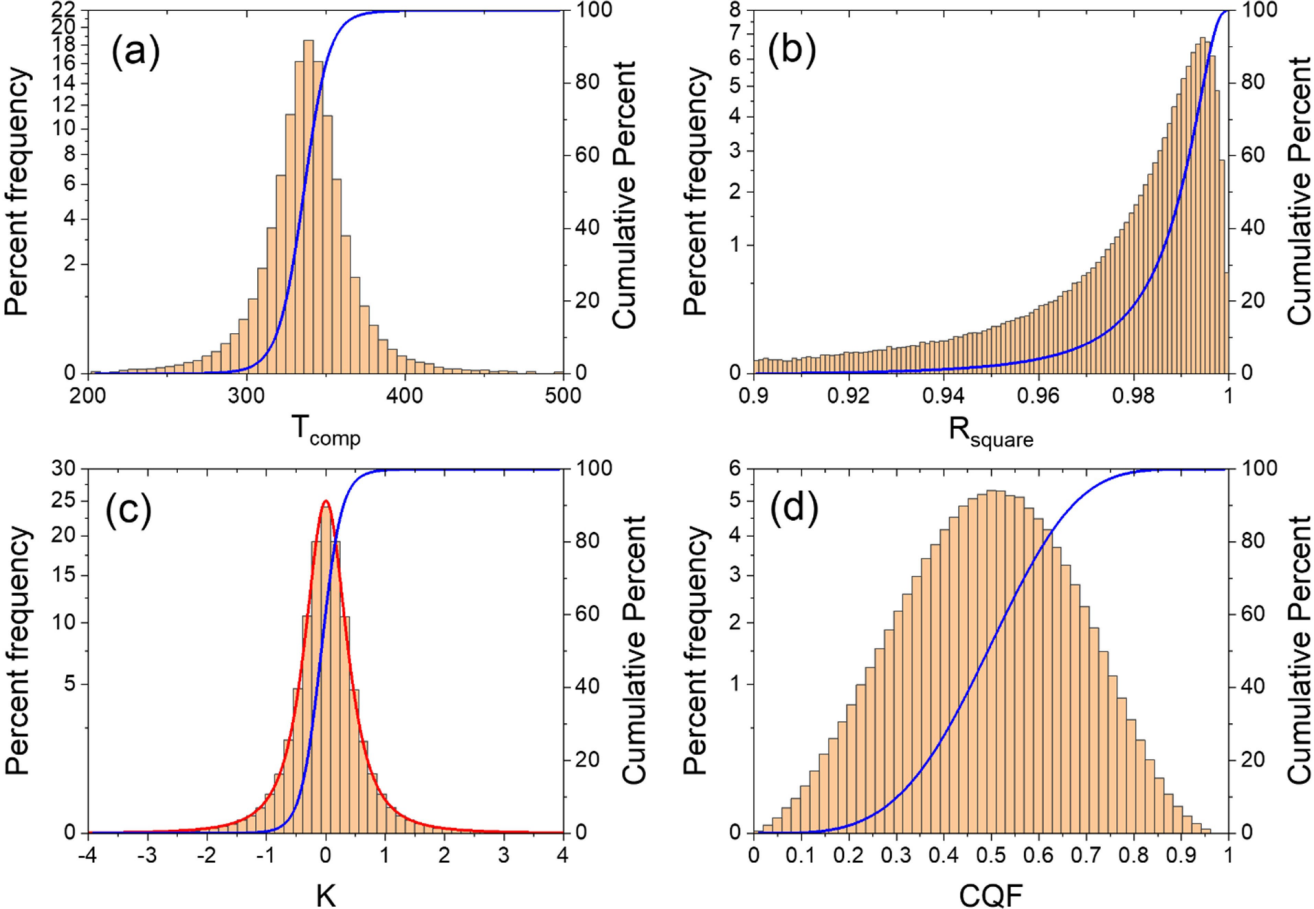
Percent frequency histograms derived from the 500000 randomly generated Van ‘t Hoff plots for *N*=8 samples for (a) the compensation temperature *T_comp_* (b) the coefficient of determination *R_square_* (c) the parameter *K* (see Eq.(8)) and (d) for the Compensation Quality Factor (see Eq.(7). The vertical square root scale is chosen to enhance the tails of the histograms. In the four panels the sum over all bins of the percent frequency is 100 % and the blue lines indicate the cumulative percent. In the histogram for *K* the red line corresponds to 25(1+3.2 K^2^)^−3.2^ and is symmetric with respect to *K*=0.

We demonstrate now that the couple (*K*, *CQF*) can be used to determine whether or not the *EEC* has a statistical origin within a chosen confidence level. For this we calculate *K* and *CQF* for a large number of simulations with randomly generated Van ‘t Hoff plots. For the ease of comprehension we describe the simulation procedure first for a specific example with *N=8* samples. Each Van ‘t Hoff plots consists of 4 data points at temperatures *T_j_* so that *T_1_=T_low_* and *T_4_=T_high_*. The two other temperatures *T_2_* and *T_3_* are such that the four *T_j_* are equidistant on a reciprocal temperature scale. For these 4 temperatures we construct random values for *LnP* so that −5<Ln*P*(*T*_low_)<0, −3.33<Ln*P*(*T*_2_)<1.67, −1.67<Ln*P*(*T*_3_)<3.33 and 0<Ln*P*(*T*_high_)<5. This means that the magnitude of the random spread is 5 at all *T_i_* . Choosing furthermore *T*_low_=300 K and *T*_high_=400 K leads to enthalpies of formation of the order of −50 kJ/moleH_2_ and entropies around −145 J/K/moleH_2_, values which are commonly observed for hydrogen absorption in metals. The harmonic mean temperature is *T*_hm_=342.85 K. For each simulation we calculate *R_square_*, *T_comp_*, *T_min_*, *K* and *CQF*. In Figure 2b the great majority, 92 %, of the simulations exhibits linear Δ*H versus* Δ*S* plots with *R_square_* >0.97 although the Van ‘t Hoff plots are **randomly generated**. This is a vivid demonstration that high *R_square_* values are easily obtained even for data that have no physical/chemical basis. Thus *R_square_* is not a good indicator of the scientific origin of an *EEC*.

In addition *T_comp_* and *T_min_* are often close to the harmonic mean temperature *T_hm_*. This is clearly put in evidence by the histogram in Figure 2a. More than 98 % of the simulations have a parameter *K* between −1 and 1. This means that for more than 98 % of the simulations *T_low_≤T_min_≤T_high_*.

As expected a very good coalescence of Van ‘t Hoff plots is not frequent. The histogram in Figure 2d shows that only 0.1 % of the simulations have *CQF*>0.9. We showed^33^ that *CQF*>0.9 is a good criterion for a significant coalescence of Van ‘t Hoff plots. A value of 0.9 implies that the smallest *LnP_Spread* is one order of magnitude smaller than the largest measured *LnP_Spread*.

On the other hand, the *CQF*‐histogram also shows that only 0.15 % of the simulations have *CQF*<0.1. This means that the probability to generate randomly a situation where all the Van ‘t Hoff plots are essentially parallel to each other is very low (This property of the Compensation Quality Factor was omitted in our previous article^[33]^).

From the expression for *CQF* in Eq.(7) one expects a correlation between *CQF* and *R_square_*. Using Eq.(6) and the definition of T* we find that

(9) $$CQF=1-\frac{1}{\sqrt{\left(\frac{R_{square}}{{1-R}_{square}}\right){\left(\frac{T_{min}}{T{}^{*}}-1\right)}^{2}+1}}\geq 1-\frac{1}{\sqrt{\left(\frac{R_{square}}{{1-R}_{square}}\right){\left(\frac{T_{hm}}{T{}^{*}}-1\right)}^{2}+1}}=1-\frac{1}{\sqrt{\left(\frac{R_{square}}{{1-R}_{square}}\right){\left(\frac{T_{high}-T_{low}}{T_{high}+T_{low}}\right)}^{2}+1}}$$

**Figure 3:**
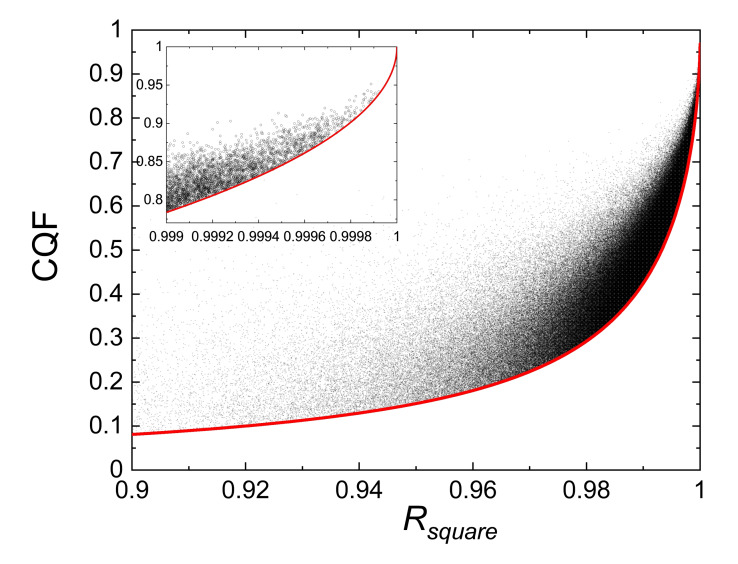
The correlation between *CQF* and *R_square_* derived from the 500000 randomly generated Van ‘t Hoff plots for *N*=8 samples is highlighted for large values of *R_square_*. The red curve is the minimum *CQF* value predicted by Eq.(9) for *T_low_* =300 K and *T_high_* =400 K. The inset is a blow‐up of the top‐right part of the graph.

The existence of a *R_square_* ‐dependent lower limit for *CQF* is shown in Figure 3. From the inset it is clear that in this case, extremely high values of *R_square_* are needed to obtain a *CQF*>0.9. Note, however, that that this lower limit depends strongly on the temperature interval chosen.

**Figure 4:**
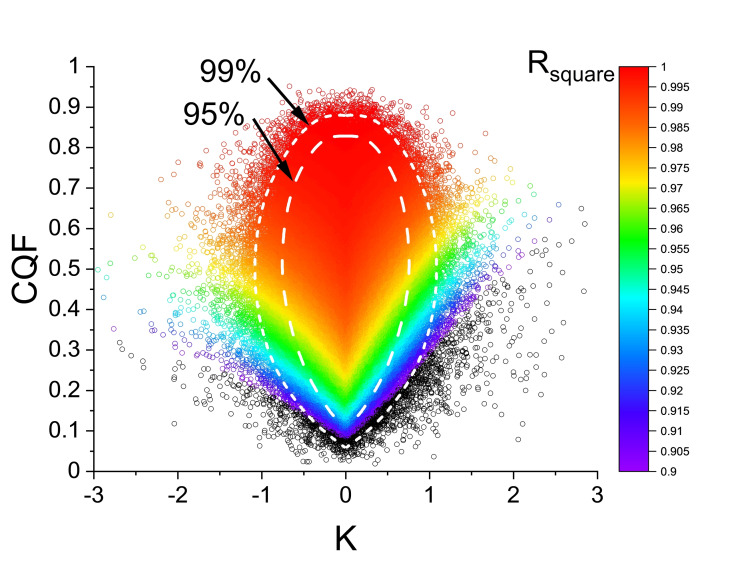
Variation of *R_square_* as a function of *K* and *CQF* for the simulations of Figure 2 (*N*=8). Each of the 500000 simulations is indicated by a coloured circle. As the great majority of *R_square_* values fall between 0.9 and 1 a black colour is assigned to the few simulations with *R_square_* <0.9. The white contours labelled 95 % and 99 % indicate the regions which contain 95 %, respectively 99 %, of all simulations. The determination of these confidence contours is described in the next section.

For a deeper insight in the relation between the three parameters *K, CQF* and *R_square_* shown in Figure 2 we indicate *R_square_* on a colour scale as a function of *K* and *CQF* in Figure 4. This graph shows vividly that good coalescence of van ‘t Hoff lines is only occurring for *R_square_* values extremely close to 1. It also shows that simulations with *CQF*>0.9 are most abundant in a region centred at *K*=0, and extend to quite low values of *CQF*.

Before describing our improved scheme, it is essential to stress that the histograms for *K* and *CQF* in Figure 2 are independent of the numerical values chosen for the simulations. This universality arises from the fact that *i)* both parameters *K* and *CQF* are dimensionless ratios and *ii)* the Van ‘t Hoff plots fitted to the random *LnP* values at the four *T_j_* are straight lines in a reciprocal temperature scale. A change in the magnitude of the random *LnP* spread (i. e. taking a value different from 5 mentioned above) has therefore no effect on *K* and *CQF*. The same is true for a choice of temperatures *T_i_* that is different from *T_low_* =300 K and *T_high_* =400 K chosen arbitrarily above.

## 4 Combined K‐CQF Method: The New 2‐Parameter Approach

**Figure 5:**
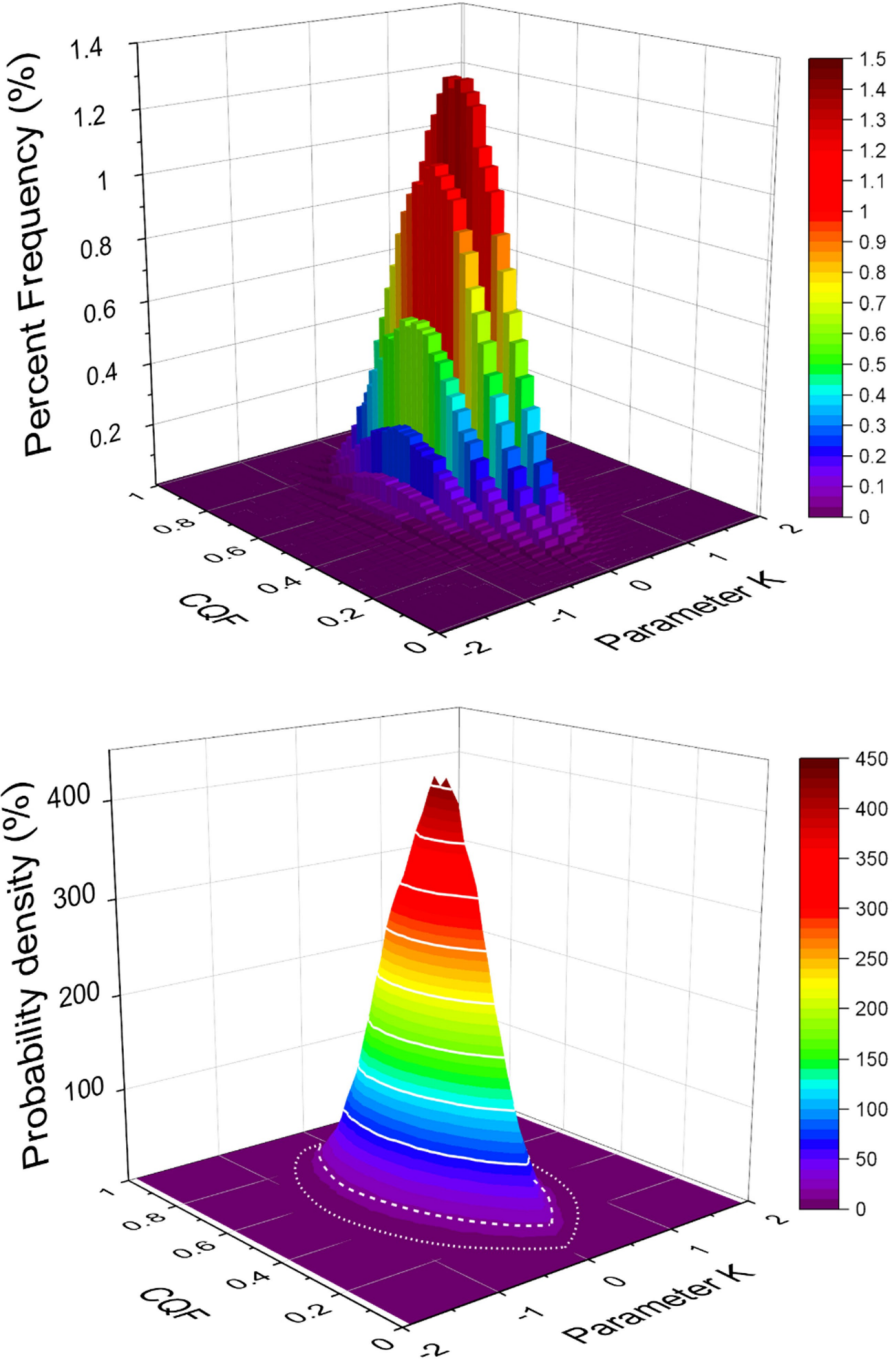
(*K,CQF*) couples obtained from 500000 simulations for *N*=8 represented as 2D‐Percent Frequency histogram (top) and 2D‐Probability Density surface (bottom). In the top panel the **sum** over all bins is 100 % while in the bottom panel the **integral** over the *K*‐*CQF* plane is 100 %. Note that the probability density surface depends **only** on the number of samples *N*. It is independent of the magnitude of the random spread in LnP and the chosen *T_low_* and *T_high_*.

As both *CQF* and *K* are universal in the sense that they both are independent of the temperature interval and the magnitude of the random *LnP_Spread* chosen for the simulations, it is interesting to construct a two‐dimensional histogram for the (*K, CQF*) pairs obtained from the same simulations as those used to generate the results in Figure 2. The 2D‐Percent Frequency histogram for (*K*, *CQF*) is shown in Figure 5. In this representation the height of each bar, the percent frequency, is defined as

(10) $$Percent\;Frequency\equiv 100\frac{Counts}{500000}$$

The **sum** over all bins of the Percent Frequency is 100 %. Although this representation is a straightforward extension of the standard *K* and *CQF* histograms in Figure 2 it has the disadvantage that the displayed percent frequencies depend on the size of the chosen bins. For the rest of this article we therefore switch to a Probability density representation where

(11) $$Probability\;Density\;\equiv \frac{Percent\;Frequency}{Bin\;area}$$

The **integral** of the Probability Density is equal to 100 %. Both the Percent Frequency histogram and the probability density surface are strongly peaked. This property is at the basis of our new verification method.

**Figure 6:**
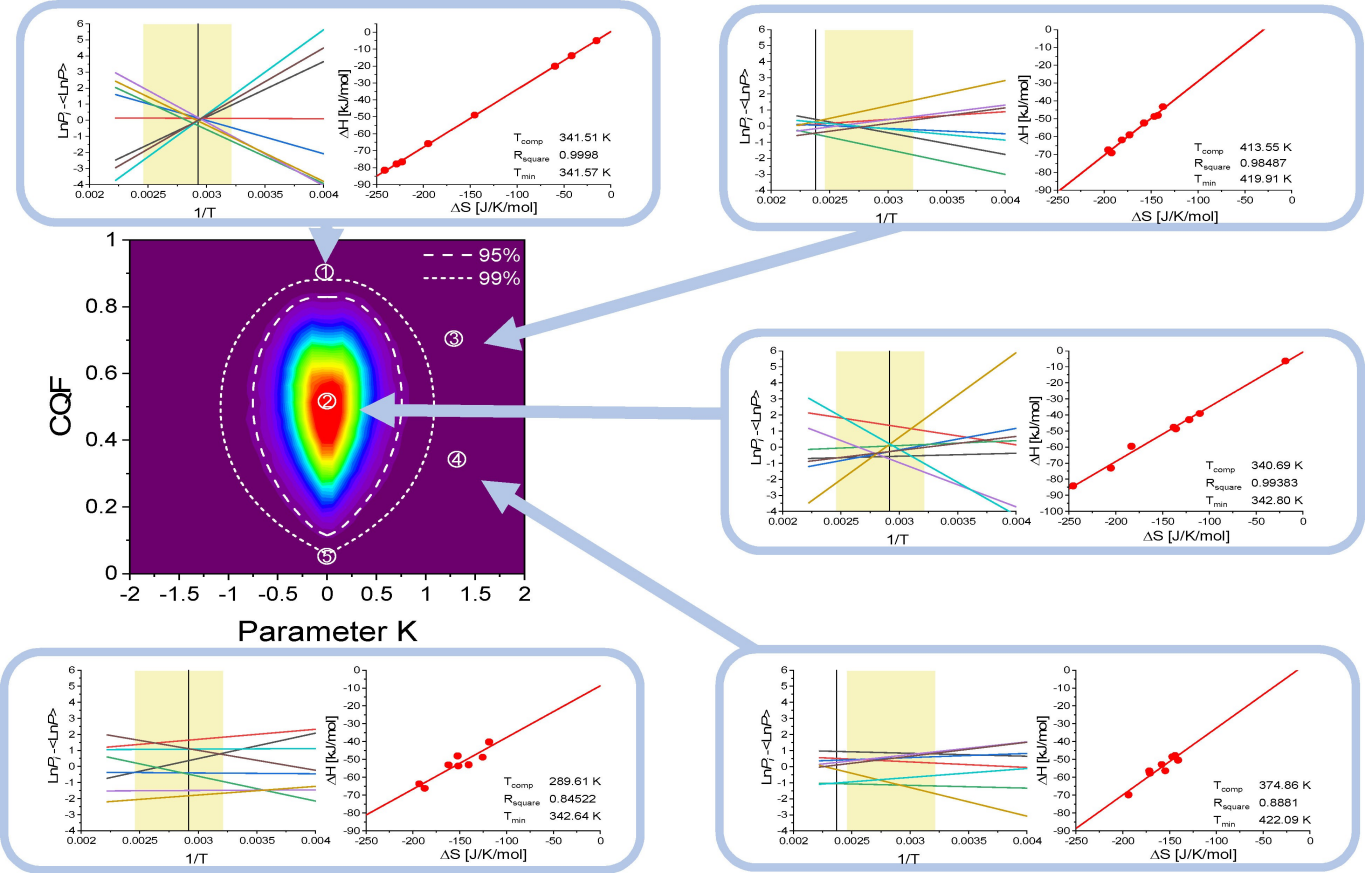
(coloured panel): Projection of the 2D‐probability density surface in Figure 5. The contours for 95 % and 99 % confidence levels are indicated as dashed and short‐dashed white curves (these white curves are the same as in Figure 5). For 5 typical (*K, CQF*) points the randomly generated Van ‘t Hoff and corresponding *ΔH* versus *ΔS* plots are shown in the 5 peripheral panels together with the values for *T_comp_, R_square_* and *T_min_*. The black vertical line indicates the position of *T_min_* where the smallest *LnP_Spread* occurs. The chosen temperature interval *T_low_* =300 K and *T_high_* =400 K is indicated as the coloured rectangle. For clarity, the Van ‘t Hoff plots are shown as *LnP_i_(T)* minus the average value *<LnP(T)&gt*; taken over the 8 values of *LnP_i_(T)* at the same temperature. The examples 1, 3, 4 and 5 correspond to situations that are rarely occurring. They are all outside the 99 % confidence contour. Example 2 is near the peak of the histogram: this situation occurs most frequently and the *R_square_* values for the *ΔH* versus *ΔS* plots is typically higher than 0.99. Even higher *R_square_* values are observed for simulations with *CQF*>0.9. In the example 1, *R_square_* =0.9998 and there is a nice coalescence of the Van ‘t Hoff plots near the mean temperature *T_hm_* =342.85 K. Very important is to mention that the data in the central coloured panel are independent of the input values used for the simulations. The results in the five panels do however depend on the choice *T_low_* =300 K and *T_high_* =400 K and the amplitude of the random *LnP_Spread*. Examples with negative K values are not shown as they are qualitatively similar to those with positive *K*.

From the 2D‐probability density in Figure 5 one can extract contours of equal probability density. Of special interest are the contours that enclose a chosen fraction of all simulations. The contours that enclose 95 % and 99 % are shown in the bottom panel of Figure 5 as dashed and dotted lines. These contours are also shown in the coloured panel of Figure 6. The surrounding panels exhibit typical Van ‘t Hoff and Δ*H* versus Δ*S* plots for 5 representative (*K, CQF*) pairs for illustration.

The use of these confidence contours follows directly from the fact that all simulations outside a chosen contour have probability densities smaller than those inside the contour. In other words, the probability that an experiment generates a (*K, CQF*) pair outside the contour is small and decreases rapidly with increasing separation from the contour. Specifically, assume that in an experiment on *N*=8 samples one has experimentally found that *K*=0.75 and *CQF*=0.3. The point (0.75, 0.3) lays outside the 95 % and inside the 99 % confidence contour for *N*=8 samples in Figure 6. One concludes that the corresponding Enthalpy‐Entropy Compensation is probably NOT due to statistical artefacts at a confidence level of ∼97 %.

**Figure 7:**
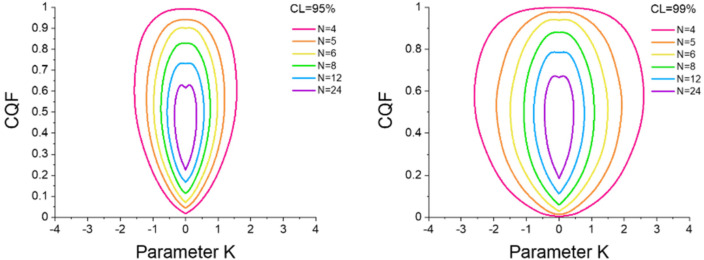
95 % (left panel) and 99 % (right panel) confidence contours for *N*=4 up to *N*=15 determined from 2D‐probability density surfaces as the one shown in Figure 5 for the case *N*=8. The green curves for *N*=8 are the same as the dashed and short‐dashed curves in Figure 5 and Figure 6.

By repeating simulations for various numbers of samples we determined the 95 % and 99 % confidence contours shown in Figure 7 for *N*=4, 5, 6, 8, 12 and 24. As the confidence contours are universal (in the sense that they depend only on the number of samples *N*) we can use them for all existing *EEC* that have been determined from Van ‘t Hoff plots of similar reactions.

The existence of confidence contours for the (*K, CQF*) couples makes a verification of *EEC* and isoequilibrium relationships extremely simple. Assuming that for a chosen system a set of *ΔH‐ΔS*‐data obtained from measurements between *T_low_* and *T_high_* are available, one needs only to:

1. Calculate the harmonic mean temperature *T_hm_*
2. Do a standard least square fit of the *ΔH* versus *ΔS* data to determine *T_comp_* and *R_square_*
3. Calculate the temperature *T_min_* at which the spread in *LnP* is smallest using Eq.(6) and the value of the *K* parameter by means of Eq.(8),
4. Calculate the Compensation Quality Factor with Eq.(7) in which T*, the temperature at which the largest *LnP_Spread* is measured is chosen in such a way that T*=*T_low_* if 1/*T*_min_ is closer to 1/*T_high_* or T*=*T_high_* if 1/*T_min_* is closer to 1/*T_low_*.
5. For a conclusion at a 95 % or 99 % confidence level check whether or not the (*K, CQF*) point lays outside the 95 %, respectively the 99 % confidence contours in Figure 7. If the couple (*K, CQF*) lays outside the CL% contour then the *EEC* is probably not of statistical origin at this CL% confidence level.

As already mentioned, the confidence contours are universal as they depend only on the number of samples. The verification of *EEC* is therefore possible without the need to run simulations for each specific data set. This is a great advantage compared to the method described in Ref. [34]

For kinetic data, the verification proceeds exactly in the same way as there is a one‐to‐one correspondence between the Van ‘t Hoff parameters and the Arrhenius parameters, Eq.(2) corresponding to

(12) $$lnk_{i}\left(T\right)=lnA_{i}-\frac{E_{i}^{a}}{RT}$$

as *k*, the rate constant of thermally activated processes, is usually well described by an Arrhenius expression $k=Ae^{-\frac{E^{a}}{RT}}$ where *E* ^*a*^ is the apparent activation energy. The slope of a plot of *E* ^*a*^ versus *RlnA* is the so‐called isokinetic temperature *T_isokin_*, which is the analogue of the compensation temperature *T_comp_* introduced in Eq.(3). As13

(13) $$T_{min}=\frac{T_{isokin}}{R_{square}}$$

the Compensation Quality Factor for kinetic data is given by the same expression as Eq.(22) with *T_comp_* replaced by *T_isokin_*, i. e.

(14) $$CQF=1-\sqrt{\frac{1-R_{square}}{\left(\frac{1}{R_{square}}\right){\left(\frac{T_{isokin}}{T{}^{*}}\right)}^{2}-2\left(\frac{T_{isokin}}{T{}^{*}}\right)+1}}$$

and the expression for the parameter *K* is the same as that in Eq.(8).

## 5 Application to Some Examples

### 5.1 Thermodynamics of Hydrogen Absorption in Palladium Nanocubes

**Figure 8:**
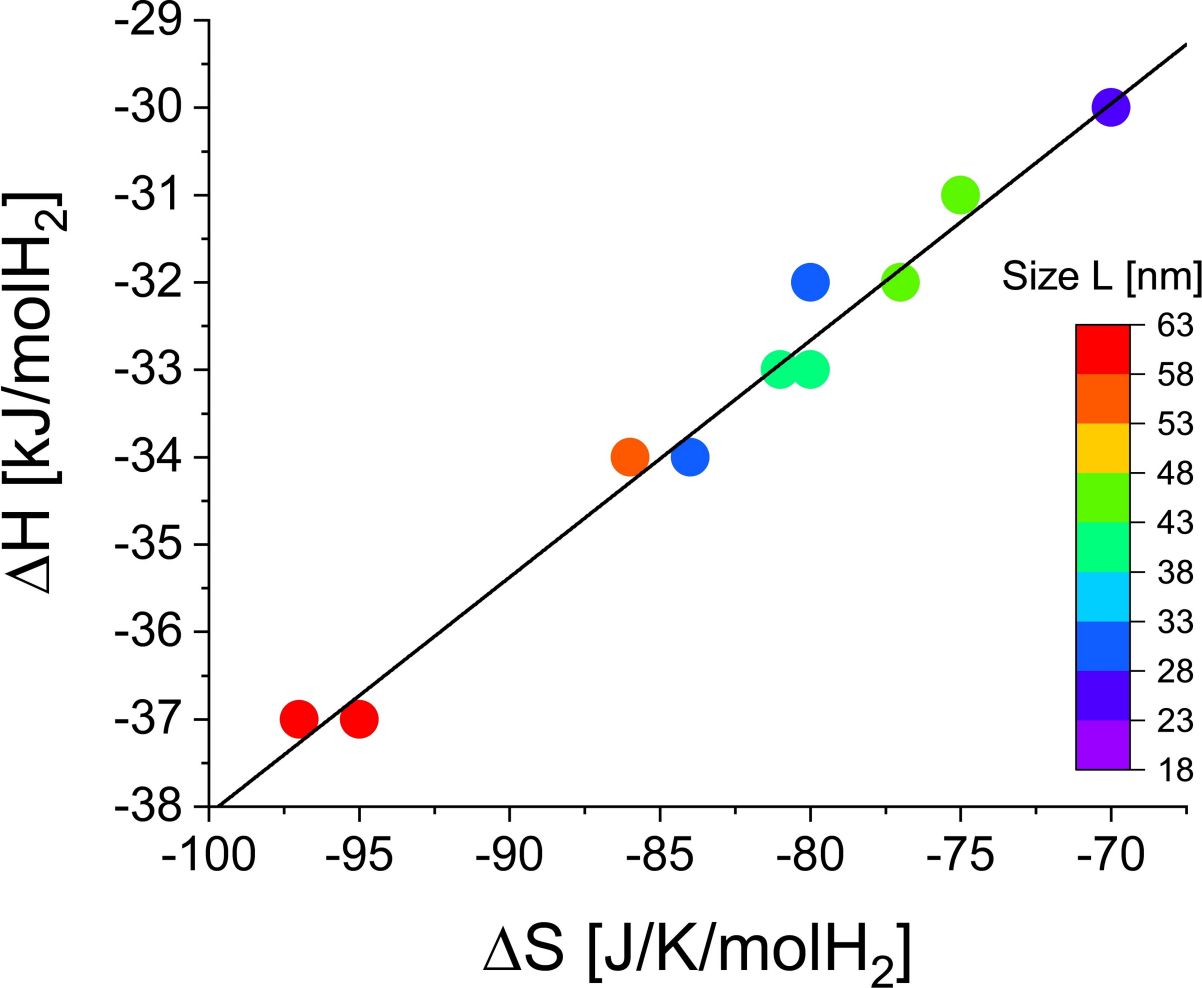
Enthalpy‐entropy plot for the hydrogen absorption in 12 Pd nanocubes of various sizes taken from Figure 4 in ref.35. The compensation temperature *T_comp_* =271 K lays well outside the experimental temperature range [303 K, 333 K]. The *R_square_* is 0.979. There is no clear relation between *ΔH* and the nanocube size.

**Figure 9:**
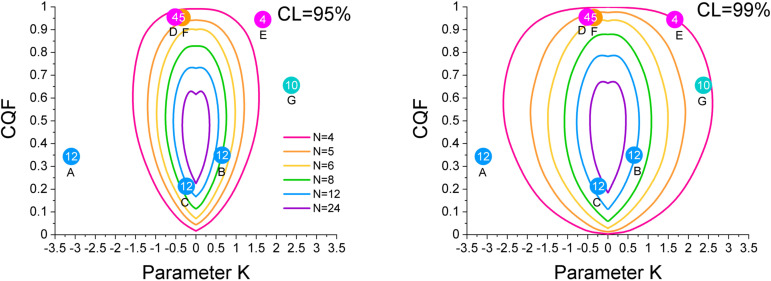
Relative position of the experimentally determined (*K, CQF*) couples and the 95 % (left) and 99 % (right) confidence contours for A) *N*=12 Pd−H nanocubes (Ref. [35]); B) *N*=12 Amid bond rotation with pyrrolidine substitution (Ref. [36]); C) *N*=12 Amid bond rotation with piperidine substitution (Ref. [36]); D) *N*=4 Wrinkled polymers at high temperatures (Ref. [37]); E) *N*=4 Wrinkled polymers at low temperatures(Ref. [37]); F) *N*=5 Ru catalysed Ammoniaborane hydrolysis (Ref. [38]); G) *N*=10 Oxidation of Cu nanoparticles (Ref. [39]).

Using plasmonic nanospectroscopy, Syrenova et al.^[35]^ made a detailed study of hydride formation thermodynamics in individual Pd nanocrystals of different size and shape and found an *EEC* with a compensation temperature *T_comp_* =289 K. As the influence of sample shape is difficult to quantify we consider here only their data for hydrogen absorption in nanocubes. The *ΔH‐ΔS* experimental data listed in Figure 4 of Ref. [35] exhibit a linear relation between enthalpy and entropy shown in Figure 8 with a compensation temperature *T_comp_* =271 K. From these *ΔH‐ΔS* data we find the couple *K*=−3.096 and *CQF*=0.3437, which is well outside the 99 % confidence contour in Figure 9. One must therefore conclude that although the coalescence of the Van ‘t Hoff plots is low ( *CQF*=0.3437 means that there is only a ratio of 2 : 3 between the smallest and largest *LnP_Spread*) the origin of the Enthalpy‐Entropy Compensation is not due to a statistical effect. This is at variance with the conclusion in our previous article.^[33]^ It shows that it is not sufficient to look only at the value of *CQF* to draw a conclusion about the origin of an *EEC*. Taking *K* into account is essential for a correct analysis.

### 5.2 Partial Amid Bond Rotation

In a recent article Guerra et al^[36]^ investigate Enthalpy‐Entropy Compensation in Partial Amide Bond Rotation using N,N‐Diethyl‐m‐toluamide (DEET) as a model system to exploit chemical exchange caused by restricted rotation about the amide bond. As application of our method we consider the 12 molecules with an amine substitution by pyrrolidine and also by piperidine. The enthalpy and entropy values in their Table S2 for these 12 molecules lead to *K*=0.651 and *CQF*=0.349 for the pyrrolidine and to *K*=−0.236 and *CQF*=0.213 for the piperidine. As shown in Figure 9 these (*K,CQF*) couples are just on the border of the 99 % confidence contour. The *EEC* is therefore not a statistical artefact at a 99 % confidence level. The *CQF* values are however rather low. There is thus no well‐defined isokinetic region.

### 5.3 Wrinkled polymers

By extending an in situ wrinkle relaxation method, Bhadauriya et al^[37]^ measured the relaxation of wrinkles of polymer nanocomposite films containing grafted nanoparticles. Far below the glass temperature *T_g_* the wrinkles are essentially static. With increasing temperature the wrinkles relax more and more rapidly with a characteristic relaxation time15

(15) $$\tau =\tau_{0}e^{\frac{E^{a}}{RT}}$$

**Figure 10:**
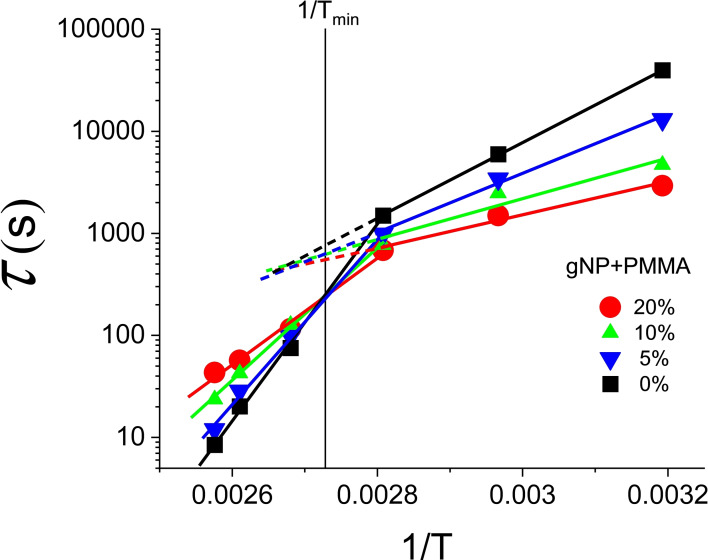
Temperature dependence of the wrinkle relaxation time τ measured by Bhadauriya et al^[37]^ for PMMA films grafted with 0, 5, 10 and 20 % concentrations of nanoparticles.

The data for *N*=4 polymethylmethacrylate films grafted with nanoparticles (PMMA‐gNP) taken from their Figure S6a are shown in Figure 10. In their article the authors forced a single Arrhenius line to the data points measured at 6 different temperatures. This procedure is questionable as the *ln*τ versus 1/*T* plots exhibit marked kinks close to 356 K. For our analysis we therefore split their data into a low temperature and a high temperature set. The high temperature set consists of 4 temperatures and the low temperature sets of 3 temperatures, the data points at T=356 K being common to both sets.

**Figure 11:**
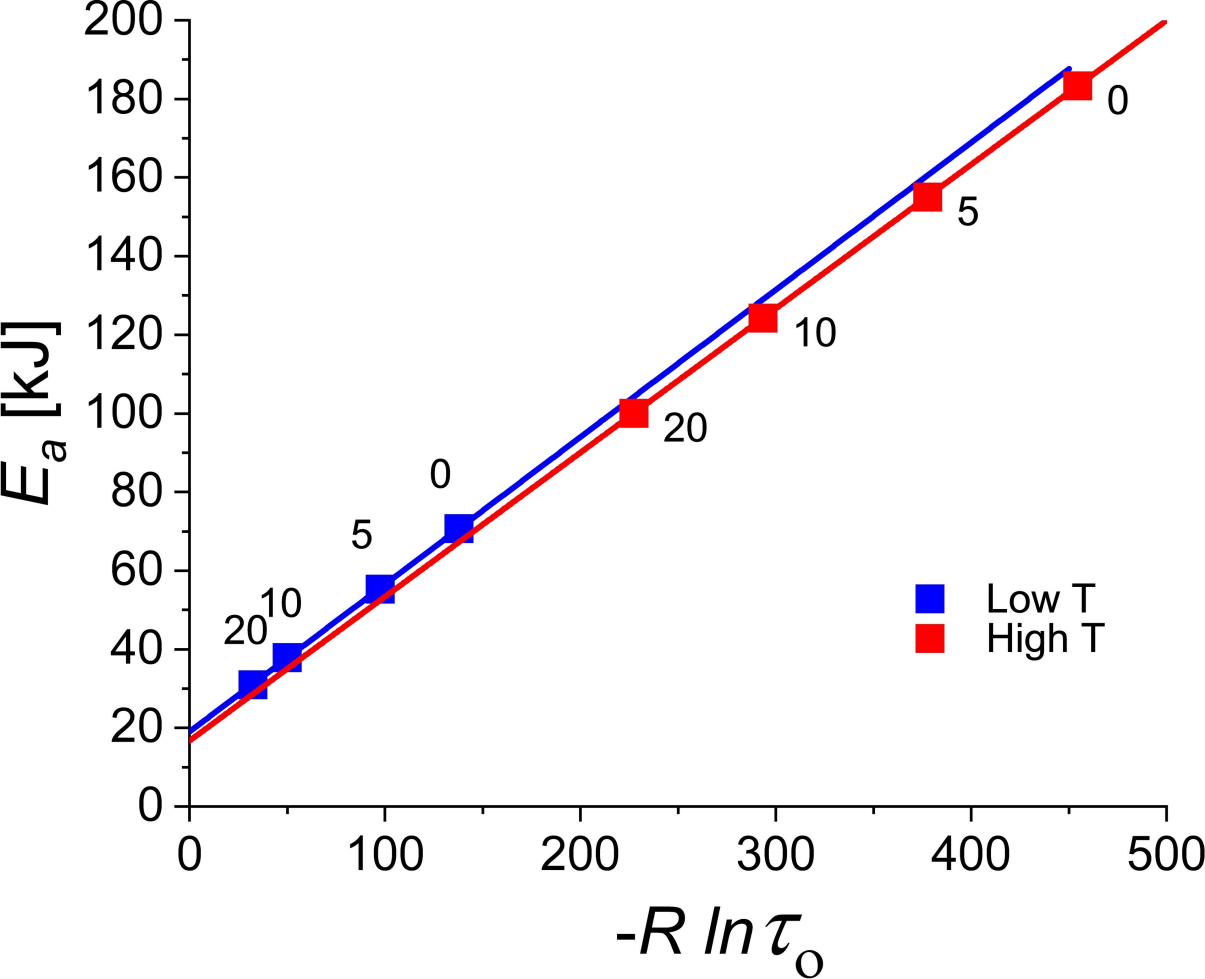
Activation energy versus prefactor plot corresponding to the Arrhenius plots in Figure 10. The slope of these lines corresponds to the isokinetic temperature *T_isokin_*. As the *R_square_* are extremely close to 1 the temperature with smallest spread in Arrhenius plots, *T_min_*, is essentially equal to *T_isokin_*. The labels indicate the concentration c in percent of the grafted nanoparticles.

From a fit to the plots of

(16) $$ln\tau ={ln\tau }_{0}+\frac{E^{a}}{RT}$$

in Figure 10 we find at low temperatures *K*=1.66 and *CQF*=0.944 and *T_isokin_* =374.69 K. The observed compensation is therefore real at a 99 % confidence level. For the high temperature set we have *K*=−0.51, *CQF*=0.955 and *T_isokin_* =366.54 K. The (*K,CQF*) couple lies inside the 95 % confidence contour for *N*=4. A detailed analysis indicates that the compensation is real at a confidence level of 87 %. Noteworthy is the fact that for both sets *T_isokin_* is the same within a few percent. This is the reason why the compensation plots in Figure 11 have essentially the same slope. At high temperatures the activation energies are systematically higher than at low temperatures (see Figure 10)

**Figure 12:**
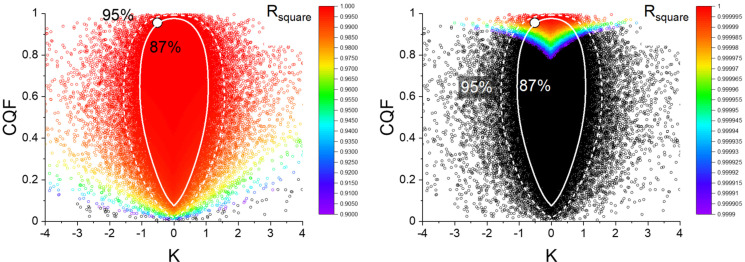
Variation of *R_square_* as a function of *K* and *CQF* for *N*=4 samples obtained from 100000 random simulations with *T_low_* =356 K and *T_high_* =388 K. The colour scale for *R_square_* in the left panel is the same as in Figure 4. In the right panel the colour scale is specially chosen to put the simulations with very high *R_square_* in evidence The white circle represents the experimental (*K, CQF*) couple obtained from the Arrhenius analysis of the high temperature data in Figure 10. A large fraction of simulations, I.e. approx. 12.5 % simulations have *R_square_* >0.9999 and still 1.4 % have *R_square_* >0.99999.

For both lines the coefficient of determination is extremely close to 1. At low temperatures *R_square_* =0.99988 and at high temperatures 0.99999. At first sight the very high value of 0.99999 for the high temperature set seems to contradict our conclusion about the (relatively low) confidence levels (87 %) derived from the position of the (*K*, *CQF*) couples for wrinkled polymers in Figure 9. To illustrate that there is in fact no contradiction we generated the *K*, *CQF* plot of 100000 random simulations for *N*=4 samples with T_low_=356 K and T_high_=388 K shown in Figure 12. The (K, *CQF*) couple for the wrinkled polymers at high temperature lies on the 87 % confidence contour in a region with *R_square_* very close to 1. Out of the 100000 simulations, 12518 simulations have *R_square_* >0.9999 and still 1410 have *R_square_* >0.99999. This shows that such high *R_square_* values can be quite common, especially in case of a small temperature range (see Eq. 9). Whenever measurements are done in a small temperature (and pressure) interval all the Van ′t Hoff lines pass through a small region of the 1/*T‐LnP* plane. As *ΔH* is the slope of these lines and *ΔS* their intercept at 1/*T*=0 there is necessarily a high correlation between *ΔS* and *ΔH*. This explains *R_square_* is not a good parameter to verify the validity of compensation effects.

### 5.4 Ruthenium‐Catalyzed Ammonia Borane Hydrolysis

In their investigation of metal‐catalyzed hydrolysis Ma and Na^[38]^ focus their attention on the influence of the compensation effect on the catalytic release of hydrogen stored in ammonia borane. Using face‐centred cubic packed Ruthenium nanoparticles supported on layered double oxide nanodisks they found an isokinetic temperature at *T_isokin_* =290.65 K. For hydrogen generation below *T_isokin_* they show that the turnover frequency of the reaction can be maximized by reducing the size of Ru nanoparticles, which increases the fraction of edge and corner atoms and lowers the activation energy. From the Arrhenius plots of *N*=5 samples in their Figure S2 we find *K*=−0.342 and *CQF*=0.953. This (*K, CQF*) couple lays outside the 95 % confidence contour and close to the 99 % confidence contour. We conclude therefore that their kinetic compensation is a real effect at a confidence level slightly lower than 99 %.

### 5.5 Oxidation of Cu Nanoparticles

**Figure 13:**
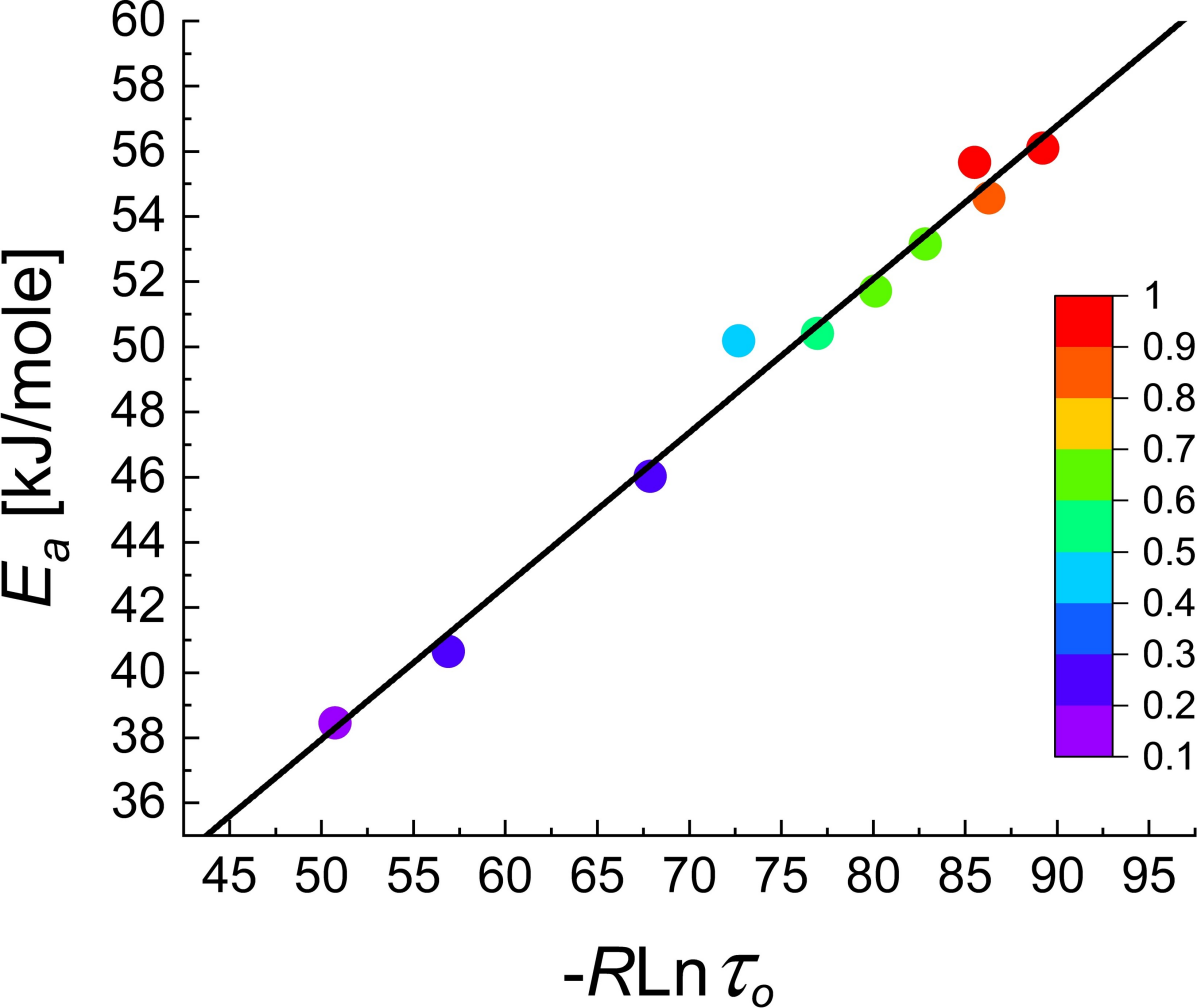
Compensation between activation energy *E_a_* and *lnτ_o_* of Cu nanoparticles with increasing extent of oxidation from 0.15 to 1 as determined by Albinsson et al ^39^

In their investigation of ∼30 nm sized Cu nanoparticles Albinsson et al^[39]^ find that the Arrhenius parameters for oxidation significantly depend on the extent of oxidation of these particles. They exhibit a distinct compensation effect between the apparent activation energy *E_a_* and the pre‐exponential factor τ_o_ as a function of the oxidation time τ as shown in Figure 13.

From the values of these two parameters given in their Figure 6a for 10 oxidation fractions we find *K*=2.376 and *CQF*=0.656. This point lays far outside the 99 % confidence contour for *N*=12 shown in Figure 9. As the 99 % confidence contour for *N*=10 lays inside that for *N*=12 we conclude that their compensation effect with *T_isokin_* =471.0 K is physical.

## 6 Conclusions

We presented an objective criterion to determine the possibility for a physical basis of the enthalpy‐entropy compensation effect. The Combined *K‐CQF* method described in Section 4 makes it possible to quantify both the degree of coalescence of experimental Van ‘t Hoff (or Arrhenius) lines and to verify whether or not the Enthalpy‐Entropy Compensation is of a statistical origin at a confidence level CL%. The method is universal in the sense that the confidence contours depend only on the number of samples N and the chosen level of confidence CL%. It can handle data sets with any value of *CQF*, i. e. also data with a poor coalescence of Van ‘t Hoff (or Arrhenius) plots.

For an easy application of the Combined *K‐CQF* method to a set of *ΔH‐ΔS* data obtained from measurements between *T_low_* and *T_high_* with a harmonic mean temperature *T_hm_*, one needs only to do a least square fit of the *ΔH* versus *ΔS* line to determine the compensation temperature *T_comp_* and the coefficient of determination *R_square_*. From these parameters one directly obtains the temperature *T_min_=T_comp_/R_square_* at which the spread in *LnP* is smallest, and the two key parameters *K* and *CQF* can be simply calculated. Finally, the position of the (*K, CQF*) point with respect to the confidence contours is used to verify the non‐statistical origin of the *ΔH‐ΔS* compensation.

As the Combined *K‐CQF* method does not require more than a standard linear fit to the *ΔH‐ΔS* data and a comparison with universal confidence contours, it provides a very efficient way to easily analyse any published enthalpy‐entropy data. *CQF* provides a clear indication for the existence of an iso‐equilibrium, while *K* indicates whether it falls within or outside the range of measurements. The method is applicable in the same way to the Arrhenius plots of kinetic studies.

Finally, it is important to realize that the Combined *K‐CQF* method considers only the statistical aspects of *ΔH‐ΔS* data. No use is made of the dependence of *ΔH* and *ΔS* on a characteristic of the investigated samples, for example, the composition of alloys, the size of the nanocubes, the geographic location of the habitat of fishes, the degree of oxidation etc. Furthermore, the physical nature of the compensation is not discussed here but includes sources of systematic errors in addition to more fundamental reasons.^[34]^

## Note

The authors declare no competing financial interest.

## Author Contributions

RG has conceived the analysis and both authors have jointly written the present manuscript.

## Conflict of interest

The authors declare no conflict of interest.
